# Is there a “price that’s right” for at-home COVID tests?

**DOI:** 10.1371/journal.pone.0282043

**Published:** 2023-03-13

**Authors:** Rebecca Portney Kirby, Michal Maimaran, Kara M. Palamountain

**Affiliations:** Kellogg School of Management, Northwestern University, Evanston, Illinois, United States of America; Jiangsu University, CHINA

## Abstract

The COVID-19 pandemic has impacted the daily lives of individuals across the world as multiple variants continue introducing new complexities. In December 2021, which is when we conducted our study, pressure to resume the normalcy of daily life was mounting as a new variant, Omicron, was rapidly spreading. A variety of at-home tests detecting SARS-CoV-2, known to the general public as “COVID tests,” were available for consumers to purchase. In this study, we conducted conjoint analysis utilizing an internet-based survey by presenting consumers (n = 583) with 12 different hypothetical at-home COVID test concepts that varied on five attributes (price, accuracy, time, where-to-buy, and method). Price was identified as the most important attribute, because participants were very price sensitive. Quick turnaround time and high accuracy were also identified as important. Additionally, although 64% of respondents were willing to take an at-home COVID test, only 22% reported they had previously taken the test. On December 21, 2021, President Biden announced the U.S. government would purchase 500 million at-home rapid tests and distribute them for free to Americans. Given the importance of price to participants, this policy of providing free at-home COVID tests was directionally appropriate.

## Introduction

The COVID-19 pandemic has impacted the daily lives of individuals across the world as multiple variants continue introducing new complexities [1]. In December 2021, which is when we conducted our study, a new variant, Omicron, drove a dramatic increase in cases [2]. With Americans looking to return to normalcy, testing for the virus remains a key component. To assist in the management of the COVID-19 pandemic, the National Institutes of Health launched the Rapid Acceleration of Diagnostic RADx® Tech program in April 2020 to make testing for active infection of SARS-CoV-2 widely available. By supporting the development and scale up of point-of-care and home-based tests, these programs increased testing capacity in the U.S. by billions and the first over-the-counter test for use at home was approved [3].

Because the development of at-home tests for infectious diseases such as SARS-CoV-2 is a relatively new phenomenon, we set out to identify consumer sentiment toward rapid testing that would provide users the opportunity to test on their own. At-home self-testing using rapid tests allows for expanded testing capacity and improved convenience, because consumers can test within the comfort of their home and limit virus exposure.

At-home rapid testing has quickly become a key consideration from a policy perspective as well. In December 2021, President Biden announced the U.S. government would purchase 500 million at-home rapid tests and distribute them for free to Americans [4]. However, it was not until January 20, 2022, that every U.S. household could order up to four rapid tests through COVIDTests.gov which would be sent directly to their home address and distributed through the United States Postal Service (USPS) free of cost [5].

Such an effort to make tests available for free relies on the assumption that price is an important barrier for people to purchase the tests. To examine the validity of this assumption, we conducted quantitative research to (1) understand how consumers evaluate at-home testing options and (2) uncover the relative importance of the various features related to testing, such as price, accuracy, and turnaround time.

Thus, we conducted a quantitative survey between December 10 and 23, 2021. During this time, a testing deficit existed because the highly transmissible Omicron variant had created a surge in demand as Americans were traveling and gathering for the holidays, but tests were in short supply [6, 7]. As of December 10, 2021, 13 rapid at-home SARS-CoV-2 tests were available on the market [8]. These tests varied across attributes including price, accuracy, time to result, availability of purchase (e.g., online vs. in-store), and method of administration (e.g., nasal vs. saliva).

## Methodology

### Study design

This study consisted of an internet-based survey that was reviewed and approved by Northwestern University IRB (STU00215811). Qualtrics recruited the participants from their existing panels and compensated them. Participants read through the internet-based survey consent form online and had to provide written consent by clicking a box that read “Yes, I consent to participate” prior to initiating the survey.

### Sample

A total of 583 participants completed the survey. Eligibility for enrollment in the survey required the participant be located and reside within the U.S. and be over 21 years of age. We collected the following background variables to profile the population: age, gender, race, urbanicity, state residence, household income, and education attainment. Participants were also asked whether they had previously taken an at-home COVID test, their experiences with their most recent at-home COVID test, and their willingness to take an at-home COVID test.

### Conjoint analysis

The main portion of the study consisted of the conjoint task in which participants rated their likelihood of purchasing and using12 hypothetical concepts for at-home tests. Conjoint analysis (sometimes referred to as “trade-off analysis”) measures the relative value consumers assign to various product features and the importance of different product attributes [9–13]. Conjoint analysis allows for the estimation of a consumer’s value system, which specifies how much value or “utility” a consumer places on each level of a number of features. As a first step, consumers evaluate several profiles (which are combinations of attribute levels). Based on the consumer’s valuation of the profiles presented, the relative preference for each level of a particular attribute is deduced and assigned a utility value. The higher the utility value, the more preferable the attribute level. From these utilities, the relative importance of each attribute can be calculated: the bigger the gap between the utility assigned to the least preferred level and the most preferred level, the more important the attribute is.

In our research, we focused on five attributes to describe the at-home tests: price (the total out-of-pocket cost the user pays for the test), accuracy (likelihood of providing the correct result), time (how quickly the sample is collected and processed), buy (where the user obtains or purchases the test), and method (how the test sample is collected). Each attribute had two to four levels (Table 1).

**Table 1:** List of attributes and levels used in the conjoint-analysis exercise.

*Attribute*	*Description*	*Levels*
1. Price	Total cost of the test, including shipping and consultation, if applicable, that you would pay for out-of-pocket	(a) $5 (b) $15 (c) $25 (d) $35
2. Accuracy	Likelihood that the test a person takes provides the correct result	(a) 85% (b) 98%
3. Time	Where and how quickly sample is collected and processed	(a) 15 minutes to test result (b) Sample mailed in, 48–72 hours to test result
4. Method	How test sample is collected	(a) Saliva (b) Nasal
5. Buy	How a user obtains the test	(a) In store (b) Online

Given that consumers cannot meaningfully evaluate all 64 possible combinations of profiles that can be created from these attribute levels, we used a fractional-factorial-design algorithm to generate 12 unique profiles (Table 2). This algorithm ensured that even though consumers did not see all possible combinations, we were still able to estimate how much the consumer valued each level of each attribute. We presented participants with these 12 different profiles (in random order) and told them, “Pretend you are looking to purchase a single at-home COVID test that you would pay for in full.” We then asked them to rate each option based on the likelihood of purchasing and using the test, on a scale of 0–100.

**Table 2:** Twelve conjoint profiles.

PROFILE	PRICE	ACCURACY	TIME	METHOD	BUY
**(1)**	$5	98%	48	Saliva	Online
**(2)**	$15	85%	15	Saliva	Online
**(3)**	$35	85%	48	Nasal	Online
**(4)**	$15	85%	48	Nasal	In store
**(5)**	$15	98%	48	Saliva	In store
**(6)**	$25	98%	15	Nasal	In store
**(7)**	$15	98%	15	Nasal	Online
**(8)**	$5	85%	15	Nasal	In store
**(9)**	$25	98%	48	Nasal	Online
**(10)**	$35	98%	15	Saliva	In store
**(11)**	$25	85%	15	Saliva	Online
**(12)**	$25	85%	48	Saliva	In store

## Results

### Relationship between demographic variables, taking the test, and willingness to take the test

A breakdown of participants based on the demographic profile information collected is presented in Table 3. Of the 583 participants, only 22% (n = 130) had taken an at-home COVID test as of December 2021. In a series of crosstabs analysis, we found statistically significant relationships between taking the test and the demographic variables of age, gender, income, education, and location.

**Table 3:** Participant demographic profile information and significant relationships with testing and willingness to test.

*Demographic Profile*	*n (sample size)*	*Total (%)*	*Significant relationship with Tested* *	*Significant relationship Willing to Test*
***Age***				
*21–34*	*207*	*35*.*5%*	*Obs*: *68 (52*.*3%)* *Exp*: *46*.*2 (35*.*5%)* *Z2 = 10*.*3*, *p <* .*005*	*Ns*
*35–54*	*225*	*38*.*6%*	*Ns*	*Ns*
*55+*	*151*	*25*.*9%*	*Obs*: *8 (6*.*15%)* *Exp*: *33*.*7 (25*.*9%)* *Z2 = 19*.*6*, *p <* .*005*	*Obs*: *41 (37*.9%)** Exp: *28 (25*.*9%)* *Z2 = 6*.*1*, *p <* .*05*
***Gender***				
*Female*	*371*	*63*.*6%*	*Ns*	*Ns*
*Male*	*208*	*35*.*7%*	*Ns*	*Ns*
*Non-binary / third gender*	*4*	*0*.*7%*	*Ns*	*Ns*
***Race***				
*Black or African American*	*82*	*14*.*1%*	*Ns*	*Ns*
*Other (includes Asian or Pacific Islander*, *American Indian*, *or Alaskan Native)*	*80*	*13*.*7%*	*Ns*	*Ns*
*White*	*421*	*72*.*2%*	*Ns*	*Ns*
***Ethnicity***				
*Hispanic*	*84*	*14*.*4%*	*Ns*	*Ns*
*Not Hispanic*	*499*	*85*.*6%*	*Ns*	*Ns*
***Education***				
*High school degree*	*241*	*41*.*3%*	*Obs*: *30 (23*.*1%)* *Exp*: *53*.*7 (41*.*3%)* *Z2 = 10*.*5*, *p <* .*05*	*Ns*
*Bachelor’s degree*	*194*	*33*.*3%*	*Obs*: *67 (51*.*5%)* *Exp*: *43*.*3 (33*.*3%)* *Z2 = 13*.*0*, *p <* .*005*	*Ns*
*Master’s or above*	*81*	*13*.*9%*	*Ns*	*Ns*
*Other*	*67*	*11*.*5%*	*Ns*	*Ns*
***Income***				
*Less than $25*,*000*	*131*	*22*.*5%*	*Obs*: *14 (10*.*8%)* *Exp*: *26*.*8 (22*.*5%)* *Z2 = 18*.*5*, *p <* .*005*	*Obs*: *35 (32*.4%)** Exp: *24*.*3 (22*.*5%)* *Z2 = 4*.*7*, *p <* .*05*
*$25*,*000–$49*,*999*	*153*	*26*.*2%*	*Ns*	*Ns*
*$50*,*000–$99*,*999*	*179*	*30*.*7%*	*Ns*	*Ns*
*$100*,*000 and above*	*120*	*20*.*6%*	*Obs*: *49 (37*.*7%)* *Exp*: *29*.*2 (20*.*6%)* *Z2 = 7*.*9*, *p <* .*05*	*Ns*
***Location***				
*Rural*	*116*	*19*.*9%*	*Obs*: *9 (6*.*9%)* *Exp*: *25*.*9 (19*.*9%)* *Z2 = 11*.*0*, *p <* .*005*	*Obs*: 33 (33%)*** Exp: *19*.*7 (19*.*9%)* *Z2 = 9*.*0*, *p <* .*05*
*Suburban*	*258*	*44*.*3%*	*Obs*: *42 (32*.*3%)* *Exp*: *57*.*5 (44*.*3%)* *Z2 = 4*.*2*, *p <* .*05*	*ns*
*Urban*	*209*	*35*.*8%*	*Obs*: *79 (60*.*8%)* *Exp*: *46 (35*.*8%)* *Z2 = 22*.*5*, *p <* .*005*	*ns*

* % of the total who tested (N = 130)

** % of those who said they were unwilling to test (N = 108)

*** % of those who said they were unsure if they were willing to test (N = 99)

Obs = observed frequency

Exp = expected frequency. Expected frequency is calculated as follows: (row total X column total) / grand total. For example, 207 individuals were ages 21–34, and 130 reported taking the test; therefore, the expected frequency of test takers among this age group would be 207*130/583 = 46.2 and the expected percentage would be 46.2/130 = 35.5%.

Specifically, the demographic breakdown of survey respondents suggests 25.9% (≈33) of the 130 participants who had previously taken an at-home COVID test would have been ages 55+. However, in reality, the observed frequency was much lower for this age group: only eight participants (6.15%) had taken an at-home COVID test (*Z*^2^ = 19.6, *p* < .005). Similarly, the expected frequency of participants ages 21–34 who had taken a test should be consistent with the demographic participation breakdown of 35.5% (≈46) of participants. However, the observed frequency was that 52.3% (≈68) of participants had taken an at-home COVID test (*Z*^2^ = 10.3, *p* < .005). Thus, younger participants (ages 21–34) were over-represented and older participants (55+) were under-represented among those who had taken an at-home COVID test.

Following the same logic, we found that men were over-represented among those who had taken an at-home COVID test (46.9% vs. expected 35.7% based on survey demographics; *Z*^2^ = 4.2, *p* < .05). Regarding educational attainment, participants with a bachelor’s degree or above were over-represented (51.5% tested vs. expected 33.3%; *Z*^2^ = 13.0, *p* < .005), and participants with a high school degree or below were under-represented in those who had taken an at-home COVID test (23.1% tested vs. expected 41.3%; *Z*^2^ = 10.5, *p* < .05). Participants with an income of $100,000 or above were over-represented (37.7% vs. 20.6%; *Z*^2^ = 7.9, *p* < .05), and participants with an income of less than $25,000 were under-represented in those who had taken an at-home COVID test (10.8% tested vs. expected 22.5%; *Z*^2^ = 18.5, *p* < .005), Furthermore, urban respondents were over-represented (60.8% tested vs. expected 35.8%; *Z*^2^ = 22.5, *p* < .005), and suburban and rural participants were under-represented (suburban: 32.3% tested vs. expected 44.3% (*Z*^2^ = 4.2, *p* < .05); rural: 6.9% tested vs. expected 19.9% (*Z*^2^ = 11.0, *p* < .005).

We also asked participants about their willingness to take an at-home COVID test. The majority (64%, n = 376) were willing, 17% (n = 99) were not sure, and 19% (n = 108) were not willing. To examine if any significant relationships existed between willingness to take the test and demographic variables, we again conducted a series of crosstabs analysis and found significant relationships with age, income, and location. Specifically, as shown in Table 3, the demographic breakdown of participants suggests 24.9% (≈28) of the participants who indicated they were not willing to take an at-home COVID test would have been ages 55+. However, the observed frequency was 37.9% (≈41) (*Z*^2^ = 6.1, *p* < .05). Thus, older participants (ages 55+) were over-represented among those who were not willing to take the test. By contrast, older participants were under-represented among the 376 who said they *were* willing to take the test (19.6% vs. expected 25.9%; *Z*^2^ = 5.6, *p* < .05). Moreover, participants with an income of less than $25,000 were over-represented in those who were unwilling to take the test (32.4% vs. expected 22.5%; *Z*^2^ = 4.7, *p* < .05). Finally, rural participants were over-represented among the 99 who were unsure whether they were willing to test (33% vs. expected 19.9%; *Z*^2^ = 9.0, *p* < .05).

### Conjoint analysis

A key finding in conjoint analysis is understanding the part-worth utilities to identify what attributes need to be included to develop a product acceptable to consumers. Part-worth analysis allows us to estimate the relative utility a consumer is deriving from changes from one attribute level to another level. To calculate part-worth, we followed the standard approach and ran a linear regression for each participant, with the different levels as the independent variables and rating as the dependent variable. The base levels in the regression were accuracy = 85%, time = 15 minutes, method = nasal, buy = in store, and price = $5.

To understand the overall preference for the different levels, we averaged the part-worth utilities across the entire sample (Table 4 and S1 File). From there, we can calculate changes in utility at the aggregate level. For example, increasing the price from $5 to $15 would decrease utility on average by 13.16. Similarly, increasing the accuracy of the test from 85% to 98% would increase utility on average by 14.74. Changing the testing method from nasal to saliva would increase the utility by just 2.09 on average, indicating this attribute is less important to users. Specifically, changes in the levels for price, accuracy, and time were notable, whereas changes in the levels for buy and method had a smaller impact on preference. We can also identify the most preferred product as the one with the highest utility level: price = $5 (utility = 0), accuracy = 98% (utility = 14.74), time = 15 minutes (utility = 0), method = saliva (utility = 2.09), and buy = in store (utility = 0).

**Table 4:** Average part-worth values for attribute levels.

*Attribute*	*Levels*	*Average Part-worth*
1. Price	(a) $5	(a) 0.00
	(b) $15	(b) -13.16
	(c) $25	(c) -19.11
	(d) $35	(d) -22.29
2. Accuracy	(a) 85%	(a) 0.00
	(b) 98%	(b) 14.74
3. Time	(a) 15 minutes to test result	(a) 0.00
	(b) Sample mailed in, 48–72 hours to test result	(b) -11.50
4. Method	(a) Saliva	(a) 2.09
	(b) Nasal	(b) 0.00
5. Buy	(a) In store	(a) 0.00
	(b) Online	(b) -0.25

If manufacturing the most preferable product is not feasible, the part-worth analysis helps identify what attributes correspond with consumers’ overall utility to estimate willingness to pay. For example, a typical rapid test, with 85% accuracy, would cost $5. We know consumers prefer the high-accuracy test (98%), and we can estimate how much they would be willing to pay for it. Based on the part-worth analysis (using only the part-worth of the relevant attributes, i.e., price and accuracy), the utility of the $5 and 85% test is 0+0 = 0. If we increase the accuracy to 98% (part-worth = 14.74) and charge $15 (part-worth = -13.16), keeping all other features the same, consumers will be willing to pay this price, because the utility of the $15 and 98% test (14.74–13.16 = 1.58) is higher than that of the $5 and 85% test (0 + 0 = 0). However, consumers will not be willing to pay $25 (part-worth = -19.11) for this test, because the utility of the $25 and 98% test (14.74–19.11 = -4.37) is lower than that of the $5 and 85% (0 + 0 = 0). Consumers are price sensitive, and increasing the accuracy of the test is not sufficient to offset the $20 price increase from $5 to $25. It is sufficient, however, to offset the smaller $10 price increase from $5 to $15.

The analysis above suggests price is highly important. To further understand the relative importance of each attribute, we calculated the importance of each attribute using the part-worth utilities for each respondent. Again, we followed the standard approach, and for each respondent, we calculated the range of each attribute (the difference between the highest calculated part-worth and the lowest calculated part-worth). We then took the sum of these ranges, and for each attribute, divided its range by the total range to create importance weights, ranging between 0 and 1. The closer the weight is to “1” (“0”), the more (less) important the attribute is. As shown in Table 5, price was the most important attribute (with an average importance of 0.36), followed by accuracy (0.2), time (0.19), method (.13), and buy (.12) across the entire sample.

**Table 5:** Five segments emerged based on attribute importance*.

Segment	Price	Accuracy	Time	Method	Buy
Overall Importance	.36	.20	.19	.13	.12
1: Money, Money, Money (N = 247, 42%)	.53	.13	.13	.11	.11
2: Time is of the Essence (N = 134, 23%)	.24	.13	.44	.10	.10
3: Accuracy Please! (N = 126, 22%)	.24	.48	.10	.09	.09
4. Mouth or Nose? (N = 41, 7%)	.20	.09	.10	.55	.07
5. Where Can I Get It? (N = 35, 6%)	.23	.09	.09	.09	.50

^*^ Numbers show average importance of each attribute for the entire sample and for each segment.

To identify key important attributes and identify any between-consumer variation, we used these importance weights in hierarchical cluster analysis. The input for each participant was the five importance weights, one for each attribute. As an initial step, the dendrogram (S2 File) clearly suggests five clusters. Creating these five clusters revealed that the largest cluster (42%, n = 247) valued price the most (average importance of price is 0.53). The second and third largest clusters (23%, n = 134, and 22%, n = 126) respectively valued time (average importance of time is 0.44) and accuracy (average importance of accuracy is 0.48) the most. Buy and method were valued the most by only 7% and 6%, respectively, of the sample (Table 5). For the segments that valued an attribute other than price the most, price remained the second most important attribute (with average importance ranging between 0.2 and 0.24). Of note, the only statistically significant relationship between demographic variables and segments was that Black or African American respondents were over-represented in the accuracy segment (20.1% actual vs. expected 14.0%, *Z*^2^ = 3.9, *p* < .05).

To better understand the importance of price among consumers, we further tested if there are differences in the importance of price between those who were unwilling to test or were unsure if they were willing to test and those who are willing. We find that those who were unwilling to test or were unsure if they were willing to test valued the price of the test more than those who were willing to test. Specifically, the average importance of price among those who were willing to test was 0.33, compared with 0.405 among those who were unwilling (*t* = 3.35, *p* < .05) and 0.38 among those who were unsure (*t* = 2.27, *p* < .05). The average importance of price among those who were unwilling to test (0.405) and among those who were unsure (0.38) were not statically different from each other (*p* > .4). Although these findings show only correlation, and not causality, between the importance of price and willingness to test, they reinforce the idea that price is a main barrier to testing.

## Discussion

In this study, we conducted conjoint analysis to assess the value placed on 12 options for at-home COVID tests with varying levels of attributes for price, time, accuracy, method, and buy. We used linear-regression modeling to estimate how changes in the level of each attribute affected user acceptance of the at-home COVID test, estimated the value or part-worth utility placed on each level of the proposed attributes and the importance of each attribute, and quantified the size of clusters or consumer segments by attribute. This analysis allows us to understand the specific attributes and levels that would make an at-home COVID test most appealing to consumers.

1. *Price*: Price was the most important attribute with the largest cluster of consumers that value price above all other attributes. Additionally, price was the second most important attribute among all other clusters. Finally, price was even more important among those who reported they were unwilling to test compared to those who reported being willing to test.
2. *Accuracy*: Accuracy was the second most important attribute overall with the third largest cluster of consumers that value accuracy above all other attributes. Consumers valued increased accuracy (98% vs. 85%), but given their price sensitivity, their willingness to pay for more accurate tests is limited.
3. *Time*: Time was the third most important attribute overall with the second largest cluster of consumers that value turnaround time above all other attributes. Consumers valued decreased turnaround time for test result (15 minutes vs. 48–72 hours), but as with accuracy, their willingness to pay for faster tests is limited.
4. *Method*: Sample collection method was the fourth most important attribute overall with the fourth largest cluster of consumers that value sample collection method above all other attributes. Consumers slightly preferred a saliva sample collection to nasal sample collection.
5. *Where to Buy*: Where to buy the test was the least important attribute overall with the smallest cluster of consumers that value where to buy a test above all other attributes. There was a minimal difference in preference for where the test would be purchased (e.g., either online versus in-store).

We observed a notable disconnect between participants who were willing to test (64%) and the minority who had actually tested (22%). This disconnect may exist because of the gap between what consumers stated they would do and actual consumer behavior or because of the limited availability of testing in December 2021. Of note, even after the time of our study, of the 500 million free COVID-19 tests that were made available to Americans in January 2022, nearly half were unclaimed at the end of February 2022 [14]. As of May 2022, nearly 350 million free tests were delivered and on May 17, 2022, the Biden Administration announced each household was eligible to order an additional eight free at-home tests [15].

This study has limitations. First, it was conducted via online survey, asking consumers to evaluate hypothetical concepts of at-home tests, limiting the external validity of this research and our ability to know whether it accurately predicts consumers’ actual preferences. Of note, the survey was accessible to participants both via internet and cell phone service, both of which are highly prevalent in the U.S. (internet penetration: 93%; smartphone penetration: 85%) across most demographic groups, with the exception of slightly lower rates for those ages 65+, individuals with a high school diploma or less, and households earning less than $30,000 [16, 17]. Although we attempted to mirror U.S. census demographics in our survey participation, these factors could have played a role in access to the internet-based questionnaire and impacted our findings.

Additionally, the study was conducted in December 2021 in the midst of a surge of new cases due to the highly transmissible omicron variant and an increase in demand for testing around the holiday and travel season. This may have affected consumer attitudes and willingness to use at-home rapid COVID-19 tests and there is a possibility this impacted the results of the study.

Finally, given that the landscape of at-home COVID testing is rapidly evolving, some of our findings may quickly become obsolete and will require continuous evaluation. Nevertheless, this research provides a framework to assess consumers’ preferences among at-home COVID tests; it can be adjusted as the landscape of tests changes and can be extended to other contexts of at-home tests.

## Conclusion

Through our conjoint analysis study, we assessed the value placed on 12 options for at-home COVID across five different attributes: price, time, accuracy, method, and where to buy. Overall, we found that consumers are extremely price sensitive as this was the most important attribute across the sample. Secondly, consumers also value quick turnaround time and high accuracy of the test. The attributes of sample collection method and location of purchase were not as important to consumers. Taken together, the data presented here confirm an interest in consumers’ willingness to purchase and take an at-home COVID test. These insights may be valuable for product developers, researchers, manufacturers, and marketers working to develop the next generation of COVID at-home tests. In addition, the results of the conjoint analysis provide an optimal configuration of product attributes that consumers would value most in an at-home COVID test.

Although policy implications are bounded by the scope of this study, the findings suggest removing price as an obstacle to consumers may increase usage of at-home COVID testing given the price sensitivity of consumers identified in our study. However, further research should be done to understand willingness to pay for incremental tests beyond those that are subsidized, as well as to understand why nearly half of Biden’s 500 million free COVID tests were still unclaimed at the end of February 2022. Future work can also focus on whether the likelihood of having taken an at-home COVID test has increased over time, what factors influence willingness to take an at-home COVID tests, and whether the intended outcome of the policy in removing price as a barrier is working properly for the end consumer.

## Supporting information

S1 File Part-worth analysis. (DOCX)

S2 File Dendogram for segmentation analysis. (DOCX)
