# Supplementary figures and images for: Is there a “price that’s right” for at-home COVID tests?
Source: PLoS One. 2023 Mar 13;18(3):e0282043. doi: 10.1371/journal.pone.0282043 (PMC10010528; doi:10.1371/journal.pone.0282043)

**Supplemental Information 1 (S1): Part-Worth Analysis**


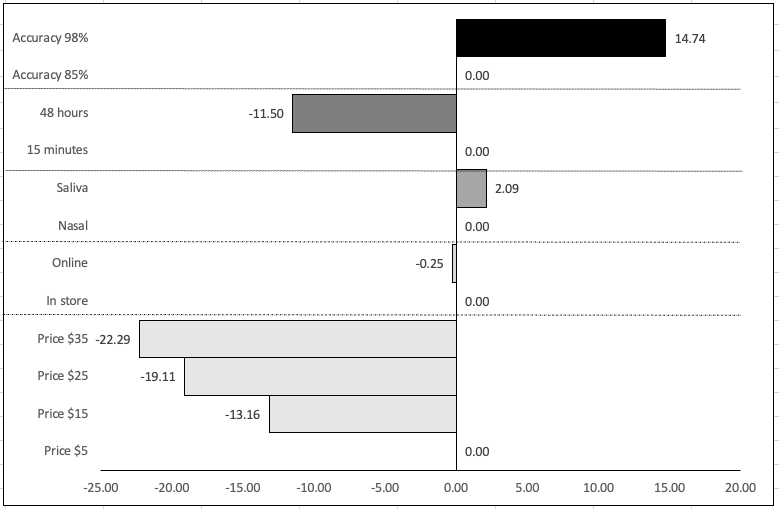

Supplement: S1 File — (DOCX) [file pone.0282043.s001.docx]
